# The latest update on the effects of COVID-19 infection in pregnancy

**DOI:** 10.18332/ejm/120973

**Published:** 2020-04-28

**Authors:** Angeliki Antonakou

**Affiliations:** 1 Department of Midwifery, Midwifery School, International Hellenic University, Thessaloniki, Greece

**Keywords:** coronavirus, pregnancy, vertical transmission, morbidity, delivery

The COVID-19 pandemic has profoundly altered the status of everyday life across the world as people are forced to social distancing and self-isolation, and to work from home via the internet. All these changes that occurred abruptly pose an immense psychological burden for all individuals and especially for pregnant women who now face their pregnancy with more anxiety and uncertainty than ever before.

The World Health Organization and several international scientific societies worldwide are in the process of constantly issuing guidelines and directives to address all COVID-19 related issues that continuously occur and which they update almost every ten days so as to incorporate newly acquired knowledge and ongoing incoming data. It is now well established that the first incidence of coronavirus 2 (SARS CoV-2) infection was detected in China on 31 December 2019. According to the European Centre for Disease Prevention and Control (ECDC), it took approximately four months for this infection to spread around the globe and to affect an estimated 1.96 million people with 123472 deaths in more than 209 countries to date^1^.

According to the latest updated guidelines from the Royal College of Obstetricians and Gynaecologists (RCOG), pregnant women do not appear more likely to contract the COVID-19 infection than the general population^2^. However, once the coronavirus has been contracted this can theoretically lead to more severe symptoms than not, since it is known that pregnancy alters the immune system and its response to viral infections. The Centers for Disease Control and Prevention (CDC) report that pregnant women manifest a more severe illness when infected with viruses from the same family as the SARS CoV-2 virus and from other respiratory tract viruses such as the influenza virus^3^. This finding of a greater severity of clinical illness in pregnant women is more prominent towards the end of pregnancy. Therefore, it cannot be ruled out that pregnant women with COVID-19 infection may potentially develop more severe symptoms such as pneumonia and marked hypoxia, in a similar manner to people who are immunosuppressed or have underlying conditions such as diabetes, cancer or chronic lung disease. The RCOG guidelines have nevertheless reported that the absolute risk of this is low^2^.

Reports from China have described that the clinical presentation of COVID-19 infection in pregnant women ranges from asymptomatic to mild flu-like symptoms with occasional atypical findings such as leucocytosis, and a higher prevalence of lung consolidation lesions in computed tomography (CT) imaging^4-6^. New emerging data from New York in the United States involving 43 pregnant women who were tested positive for COVID-19 demonstrated a similar pattern of disease severity to non-pregnant adults and was reported as mild in 86%, severe in 9%, and critical in 5% of cases^7^. In the United Kingdom, there were only two pregnant women included in the Intensive Care National Audit and Research Centre (ICNAR) report and therefore no clear conclusions can be drawn^8^. In Sweden, the Public Health Agency has reported on two pregnant women being admitted to the intensive care unit, without however any further details on the gestational week upon admission or on disease severity^9^. It is interesting that Sweden is a country that has chosen a completely different route of policy on how to deal with COVID-19 than the rest of Europe that involves less restrictions on social activities and interactions. It should be noted that to date there has been no report of maternal deaths in the published literature^2^.

The risk of intrauterine transmission of COVID-19 from a pregnant woman to her fetus has been reported to be unlikely^3^. The initial data from China have shown that there was

no evidence of the coronavirus in amniotic fluid samples, cord blood, neonatal throat swabs, placental swabs and genital fluid from COVID-19 infected mothers^10-14^. However, there is some emerging evidence at present suggesting that vertical transmission is probable2. There are reports that refer to the identification of IgM antibodies for SARS-CoV-2 in neonatal serum at birth^15,16^. Since these reports have included very small sample sizes and as it is known that IgM antibodies do not cross the placenta, this finding is most likely to represent the neonatal immune response to the in utero infection. In another study it was reported that 38 neonates who were delivered by COVID-19 positive women showed no SARS-CoV-2 infection despite the fact that some of the neonates had perinatal complications. Interestingly enough, the placenta of those neonates with perinatal complications was found to be SARS-CoV-2 positive^14^.

Another risk for pregnant women, which up to now has not been highly acknowledged, is that of maternal venous thromboembolism. It is known that pregnancy induces a hypercoagulable state that predisposes to blood clot formation. Moreover, the latest evidence from patients with COVID-19 shows that they face an increased risk of clotting^17^. Therefore, women who are both pregnant and have the COVID-19 infection have a potentially increased risk of maternal venous thromboembolism, which may be aggravated due to their reduced mobility as a result of their hospital admission or self-isolation at home^2^. Another factor that has been shown to increase maternal morbidity is smoking, which has been associated with more severe disease in COVID-19 infection. For this reason, pregnant women should be properly informed and supported in their efforts to quit smoking while pregnant^2^.

Currently, there are no data suggesting an increased risk of miscarriage or early pregnancy loss in relation to COVID-19^2^. There is scarce information about the effect of the coronavirus in early pregnancy. Previous knowledge from other types of coronavirus infections such as SARS and MERS does not demonstrate a convincing relationship between infection and the increased risk of miscarriage or second trimester loss. The risks to pregnant women appear to increase in particular during the last trimester of pregnancy^18^. There are also few reports from China where women with COVID-19 presented with preterm labour. In a review of 37 pregnant women with COVID-19 infection, it was shown that the incidence of preterm birth was 19%^19^. It is unclear though whether preterm labour in these women was iatrogenic or spontaneous^2,10^.

With regard to the mode of delivery, the initial data we have at the moment originated from China where caesarean section was seen as the method of choice as a precautionary measure to avoid perinatal transmission^5,10^. However, there has been no evidence so far of increased perinatal transmission during vaginal delivery^2,20,21^. The recent RCOG guidelines advise for the continuous intrapartum use of electronic fetal monitoring due to the high incidence of fetal distress reported in the studies from China^10,12,22^. The RCOG guidelines are explicitly clear and they state that there is no evidence to favour one mode of delivery over another. Therefore, the mode of birthing should be discussed with the woman, taking into consideration her preferences and any possible obstetric indications for intervention. The RCOG states that the presence of COVID-19 should not influence the decision for the mode of delivery unless the woman’s respiratory condition requires an urgent intervention. The Hellenic Society of Obstetric and Gynecologic Emergency has also adopted the RCOG guidelines^23^. Nevertheless, three cases of women who were positive to SARS-CoV-2 and gave birth in Greece over the last month all had a caesarean section. Unfortunately, there were no data made available on the rest of their obstetric history to justify the mode of delivery.

It is crucial to note that at present and in relation to a vaginal birth, whenever vaginal secretions were tested for COVID-19 the results have been negative^2^. Women should be informed about the risks and benefits of either choice of mode of delivery and should be fully supported in their final decision. They should also be permitted and encouraged to have a single, asymptomatic birth partner present with them during their labour and birth. We should not underestimate the importance of the presence of a trusted birth partner during labour, especially during a time of great uncertainty and fear when pregnant women face increased anxiety due to the COVID-19 itself. They may also have reduced support from their wider family and friends because of social isolation and distancing. Moreover, they may face major changes in life circumstances and great uncertainty about their financial situation. During these difficult times, midwives and health professionals in general need to support women in their choices, provide them with safe clinical care and provide guidance and increased support throughout their pregnancy and labour. It remains our duty to provide respectful maternity care to our clientele despite the changes we need to make in our everyday practice during the COVID-19 pandemic^24^.
